# Engaging Students in *PLoS Medicine*

**DOI:** 10.1371/journal.pmed.0020118

**Published:** 2005-04-26

**Authors:** The PLoS Medicine Editors

Medicine might not be the oldest profession, but the practice and the teaching of it certainly stretch far back into antiquity. In the developed world, medical schools are now thriving enterprises whose main aim remains to turn out sufficient numbers of well-trained medical practitioners. But alongside this primary purpose, medical schools have become centers of excellence for research also, mostly basic scientific research. Certainly the medical schools of the University of Cambridge and University of California at San Francisco (UCSF)—the two schools closest to the PLoS editorial offices—have large research programs.

Getting the balance right between understanding the research that will drive future medical discoveries, and providing training in clinical practice is obviously crucial in training tomorrow's doctors. However, follow-up studies of those who graduate suggest that medical schools may not be producing doctors who are happy with the profession, or who fit all the needs of medical services today. In the United Kingdom, two striking observations came out of recent surveys. One is the difficulty in recruiting doctors who want to become general practitioners, the primary care doctors who form the backbone of the UK health service. Whereas in the 1970s and 1980s 40%–50% of the qualifiers intended to enter general practice, in the 1990s the proportion was only 20%–26%. (BMJ 326: 194–195). But even more disturbing is the substantial proportion of doctors in the same surveys who indicated that they regularly consider leaving medicine altogether. Contrast these results with those of a survey from Uganda, where the survival of physicians was the most urgent concern. From a cohort of 77 doctors who graduated from the University of Makere, Uganda, in 1983, 22 had died over the following 20 years (BMJ 329: 600–601). The most important cause of death was presumed to be AIDS. But all of the surviving doctors were in some form of medical work.

What these figures show perhaps more than anything is the difficulties of generalizing about what are the top priorities for medical students across the globe. To provide a place for all students to debate the issues that matter to them, *PLoS Medicine* is launching the Student Forum, a new quarterly section written by medical students. An international team of student advisers has been elected that will help shape the content of this new section. The Student Forum recognizes the vital role that medical students have in guiding the future of the medical profession.

The first Student Forum essay brings together students from three continents: Brian Palmer of the American Medical Student Association, Amanda Wong of the European Medical Students' Association, and Mohit Singla of the Indian Medical Students' Organization. They lay out some of their concerns, such as the need for medical education to distance itself from the pharmaceutical industry and the importance of students worldwide having unfettered access to the medical literature. The students also ask questions about their training, commenting that the “reductionistic logic of medical education offers only false comfort, and only to those who content themselves with pathways and receptors.”

Perhaps surprisingly, this opinion is not far away from that of an established academic clinician and editorial board member of *PLoS Medicine*, Jonathan Rees. In another essay in this issue, Rees similarly questions the overemphasis on basic research in medical schools, posing the question, “why are our institutions not fit for the purpose of improving patients' health?”

Not an easy question to answer, but one that might also be relevant to the question of what a medical journal, especially one that wants to engage students, should publish. Should we follow Rees's advice on what the composition of a medical school curriculum should be and have no more than 20% of our articles devoted to basic scientific research as applied to medicine? In this issue the balance of our research papers comes close to this suggestion, with one paper on the differentiation of insulin-producing cells from human neural progenitors, and other papers throughout the journal on topics as diverse as climate and dengue fever, treatment of malaria, HIV genotypes, prescription practices, counterfeit drugs, Buruli ulcer, and health and human rights. But, perhaps like the medical schools, we are not paying enough attention to other topics Rees points out as important—operations research, decision-making, informatics, and economics.

In his lightning trip through medicine, *Blood and Guts: A Short History of Medicine* (W. W. Norton, 2003), the late medical historian Roy Porter wrote that “the biomedical model can be myopic, searching ever more microscopically for disease but often omitting the wider picture of populations, environments and health.” In his description of the differing opinions on the role in 19th century medical schools of the new practice of laboratory medicine, it becomes clear that the debate over what should be taught and what matters to medical students might not be new, but it is clearly as important as ever.

